# Retrospective Analysis of Surgeon-Placed Nerve Block and Indwelling Catheter in the Adductor Canal in Total Knee Arthroplasty

**DOI:** 10.7759/cureus.39833

**Published:** 2023-06-01

**Authors:** Daniel E Matthews, Robert T Rella

**Affiliations:** 1 Orthopedic Surgery, University of South Alabama College of Medicine, Mobile, USA; 2 Orthopedic Surgery, Alabama Orthopedic Sports Medicine, Daphne, USA

**Keywords:** peripheral nerve catheters, opioid use, pain management, adductor canal nerve blocks, total knee arthroplasty

## Abstract

Introduction: Total knee arthroplasty (TKA) is now being performed in the outpatient setting, and often the postoperative pain is managed with opioid analgesics. Non-opioid pain management modalities are in crucial demand, and we propose a surgical technique that can potentially result in less pain and the decrease in the use of opioid analgesia following TKA. The purpose of this study was to investigate the safety and efficacy of a novel peripheral nerve block (PNB) that includes a single injection and catheter placement for a continuous regional nerve block in total knee arthroplasty.

Methods: Fifty-six patients underwent TKA by a single surgeon utilizing the novel method. Patient-reported outcomes were entered into an outcomes database and compared to an aggregate of over 3,500 comparative TKA patients. A visual analog scale (VAS) evaluated perioperative pain. Patient perioperative opioid usage, expectations of pain control, the incidence of common side effects, and the average hospital length of stay (LOS) were collected.

Results: Compared to the aggregate of patients in the database, the patients who received the novel surgeon-placed adductor canal block (ACB) and catheter placement reported findings that suggest this technique can possibly lead to a decrease in the severity of pain in addition to a reduction in side effects and the need for opioid analgesia. LOS for these patients was short, and patient satisfaction scores were excellent for the surgeon performing this technique.

Conclusions: Using the placement technique described, surgeons can reproducibly perform a single injection of PNB and place an indwelling catheter in the adductor canal through direct visualization of the muscles that make up the borders of the adductor canal. This technique offers potential advantages over pain management modalities that can be elucidated in further studies. The power of this study is limited due to these findings having not been analyzed for statistical significance.

## Introduction

Total knee arthroplasty (TKA) is considered one of the most painful medical procedures that a patient can undergo [1-3]. Pain management in TKA patients is challenged by a postoperative imperative for early ambulation along with the public health need to reduce opioid consumption and hospital stay. Peripheral nerve blocks (PNB) address these concerns to some degree, with femoral nerve block (FNB) and adductor canal block (ACB) being the most commonly used analgesic modalities to treat post-TKA pain [4,5].

However, the optimal PNB or combination of blocks remains unclear [1,2]. There is a growing body of evidence suggesting that ACB provides analgesic effects comparable to FNB but without causing a motor blockade, which is associated with a high risk for falls [6-8]. The ACB is typically placed by an anesthesiologist prior to TKA, which inevitably leads to the consumption of valuable time and resources and increases costs. Moreover, the exact anatomical location of ACB is still debated among anesthesiologists, leading to variable pain relief. While there is no clear difference in experienced pain after TKA, FNB has been associated with motor paralysis, while ACB is a purely sensory block [2,7].

The adductor canal is a musculoaponeurotic tunnel that originates at the apex of the femoral triangle in the mid-thigh and extends distally to the adductor hiatus [9]. The canal is formed by the sartorius muscle medially, the vastus medialis muscle anteromedially, and the adductor longus and magnus muscles posteriorly, with the vastoadductor membrane (VAM) providing the “roof” [2,6,7,9].

The saphenous nerve travels between the muscles that comprise the adductor canal and is a pure sensory nerve carrying a large majority of sensory innervation from the knee [10,11]. It is also of potential benefit to the patient to perform the ACB intraoperatively, as it eliminates the need for another provider to perform an additional procedure on the patient as well as the unique opportunity to place an indwelling catheter for extended pain relief. In a prior article from the authors of this one, it has been demonstrated that the technique described here stains every nerve around the knee joint that the ultrasound-guided technique does, with the surgeon-placed ACB having the additional benefit of blocking the posterior obturator nerve that was not realized in the ultrasound-guided technique [12].

Management of post-operative pain after TKA is critically important and one of the primary reasons for the need of inpatient care after TKA. Pain control during the immediate post-operative period is also critical if patients are going to reach the rehabilitation goals required to achieve the most successful outcome [13]. Traditionally, pain management has relied heavily on parental and oral opioid analgesics [14]. Over the last decade, there has been a shift in the manner in which we manage post-operative pain, with an increase in the use of local and regional anesthesia modalities.

While surgeons have long performed local nerve blocks and joint capsular injection cocktails, regional nerve blocks have recently gained significant popularity [15]. These regional nerve blocks have traditionally been placed by anesthesia services using a nerve stimulator or, more recently, under ultrasonic guidance. While this single-injection regional (adductor canal) block can provide significant relief for the first 16-24 hours, patients can experience a significant rebound of pain as the block resolves [16]. Longer sensory anesthesia can be achieved though the placement of an indwelling catheter to provide a continuous ongoing long-acting nerve block [17].

The placement of these indwelling catheters can present challenges. Some surgeons may find that anesthesia services are either not set up to place indwelling catheters and/or not willing to provide post-operative care or monitoring of these catheters. The placement of these catheters may also present with a challenge for the anesthesia services regarding the best location for the catheter placement. If placed at the mid or low (distally) adductor canal position, the catheter can invade the sterile surgical field, or be in jeopardy when a tourniquet is used. If the catheter is placed high (proximally) in the adductor canal to avoid the tourniquet and sterile surgical field, the risk of a motor block of the femoral nerve can be realized [18]. The purpose of this paper is to describe the technique and advantages for the placement of a single injection and catheter placement in the adductor canal under the direct visualization and control of the operating surgeon. This paper also reports on the patient outcomes, safety, efficacy, efficiency, and reproducibility of this adductor canal block and catheter placed intra-operatively by the operating surgeon.

## Materials and methods

Methods

Between July and November of 2017, 56 consecutive patients who underwent primary total knee arthroplasty (TKA) performed by a single surgeon were entered into the study cohort. There were 31 females, and 25 males aged from 51 to 91 with a mean of 70.5. After informed consent, patients agreed to assist with collecting outcomes data by answering pre-operative and post-operative questions related to pain, use of opioid medications, and issues with nausea, emesis, constipation, dizziness, and drowsiness. The length of stay in the hospital was also collected. Patients were randomly entered into this study cohort as they presented consecutively through the clinic for TKA. Each of the patients had a 23-hour outpatient TKA performed. To remove selection bias, all patients were entered into the study group consecutively without any regard to co-morbidities, the extent of disease, or deformity.

Patients agreed to be entered into an email/text-based data collection program. Staff enrolled patients pre-operatively, and patients then received email and text messages to respond to questions pre-operatively and post-operatively through post-op day seven. Patient data were collected in a prospective manner and then entered into a database to compare outcomes with a database aggregate of comparative TKA patients who received a single-shot adductor canal block, placed by anesthesia, but did not receive the novel surgeon-placed block and catheter placement as described in this study. A visual analog scale (VAS) was used to evaluate perioperative pain. Patient perioperative opioid usage, patient’s expectations of pain control, the incidence of common side effects, and the average hospital length of stay (LOS) were also analyzed. 

Surgeon-placed adductor canal technique

Using a standard parapatellar, mid-vastus, or sub-vastus approach to TKA, the surgeon can readily place a catheter between the muscles that make up the adductor canal using only blunt retraction of the vastus medialis oblique muscle (VMO) without any further significant dissection. This regional nerve block and catheter placement take less than 60 seconds to perform, adding no additional time to the surgical procedure. This can be performed while the cement is curing or during the irrigation phase prior to closing if using an uncemented prosthetic. While this placement may be a slight paradigm shift for some surgeons, the medial knee joint anatomy is very familiar to all surgeons and requires a very shallow learning curve to perform.

The VMO, the adductor longus, and the sartorius are the three muscles that makeup the borders of the adductor canal. This adductor canal is easily accessed through any standard anterior surgical approach to the distal femur. The surgeon must first identify and palpate the adductor tubercle and the medial intermuscular septum, which is found as the fascia lying just anterior to the adductor longus tendon that attaches to the adductor tubercle. Two major sensory nerves, the saphenous nerve and the obturator nerve (anterior and posterior branches), are the major sensory nerves that provide sensation to the knee joint [8,13,19]. These two nerves are found within the adductor canal with the saphenous nerve exiting more proximally and the obturator nerves more distally in the canal. Each of the standard approaches for TKA in some fashion split the muscle fibers of the VMO or separate the VMO from the rectus femoris. After the placement of the TKA implants, direct access to the adductor canal can easily be obtained with the surgeon gently dissecting with the index finger by passing it parallel to the medial shaft of the femur, separating the fascia underneath the VMO. This is a blunt dissection along the medial border of the femur, staying anterior to the medial intermuscular septum.

Placing an introducer needle with a peel sheath through the skin laterally at the superior pole of the patella, the needle is placed underneath the rectus femoris and brought into this potential medial space. The surgeon is then able to pass a “soaker type” catheter through this introducer needle and then grasp the catheter to bring it into the surgical wound. After removing the peel sheath, the surgeon can grasp the catheter and pass it 8-10 inches into the adductor canal from distal to proximal between the muscles that make up the borders of the adductor canal. This reproducibly and safely places the catheter along the course of the saphenous nerve within the adductor canal. The nerve and femoral vessels are protected by the fenestrated medial intermuscular septum that runs along the nerve as it courses through the adductor canal.

## Results

**Figure 1:**
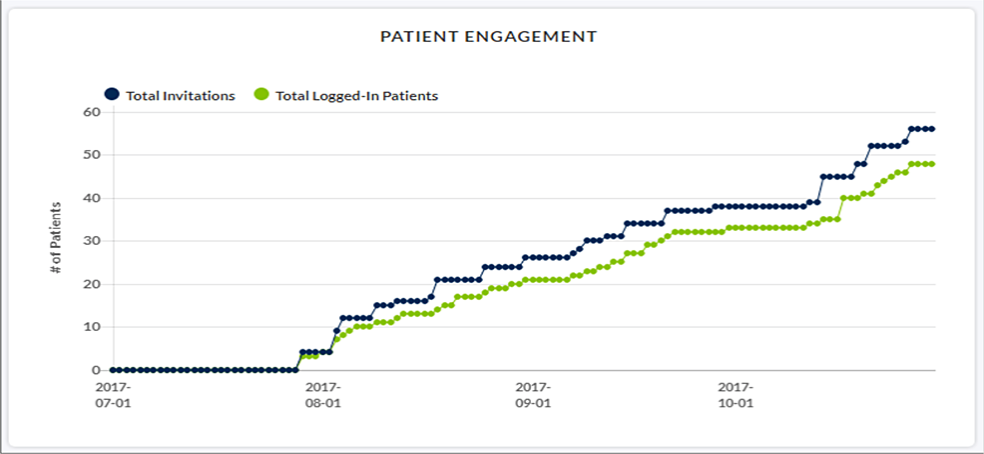
Patient engagement.

**Figure 2:**
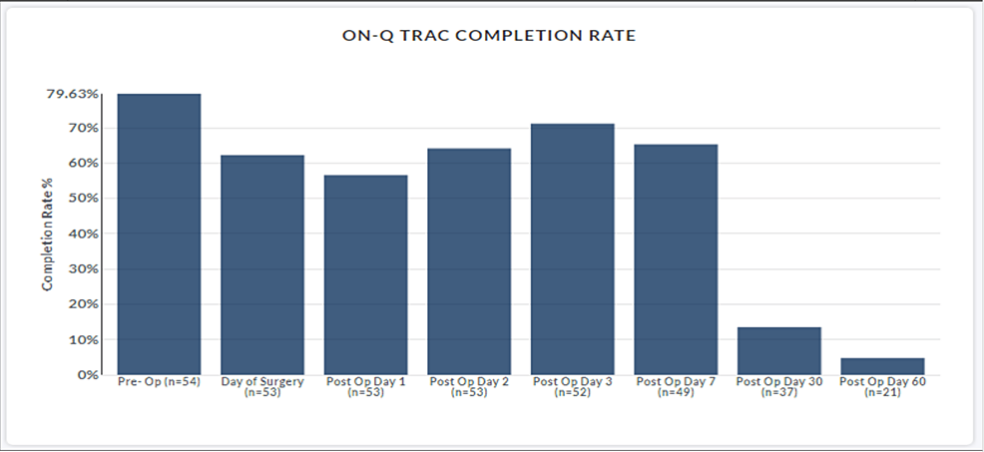
On-Q TRAC completion rate.

Fifty-six patients were prospectively entered into this original study group. Each patient agreed to complete the daily survey over the first seven post-operative days. Forty-eight patients (86%) initiated the surveys during this collection period (Figure 1). Thirty-six patients (65%) completed the entire survey (Figure 2).

**Figure 3:**
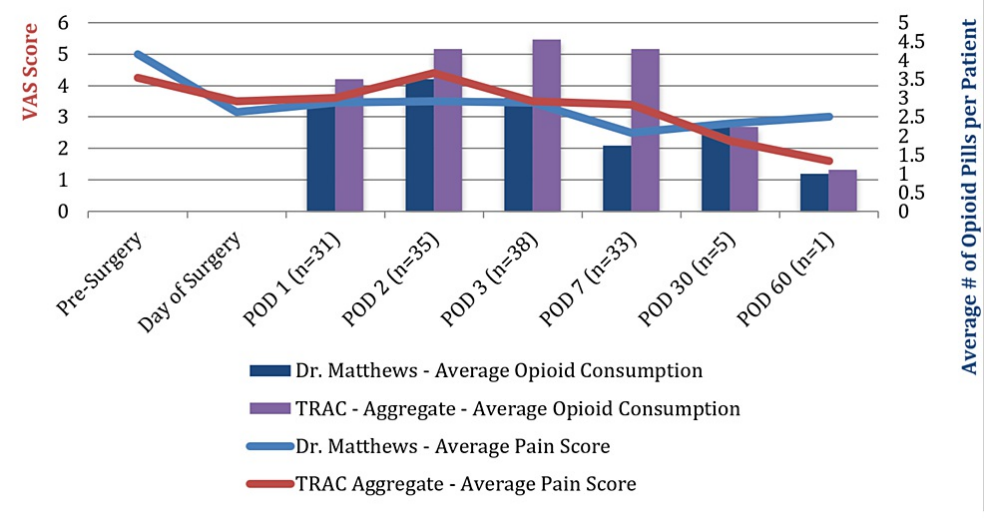
VAS pain average with opioid consumption-knee procedures. VAS: visual analog scale.

**Figure 4:**
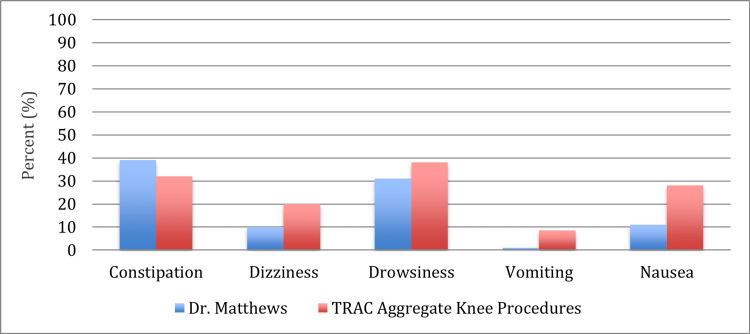
Percentage of patients reporting adverse effects.

Overall, when compared to the aggregate database, patients in this study had a 24% reduction in total opioid pills used (POD 1-7), 25% reduction in pain on the VAS (3.9 to 2.9) (Figure 3), 50% reduction in dizziness, 19% reduction in drowsiness, 88% reduction in vomiting, and 62% reduction in nausea (Figure 4).

**Figure 5:**
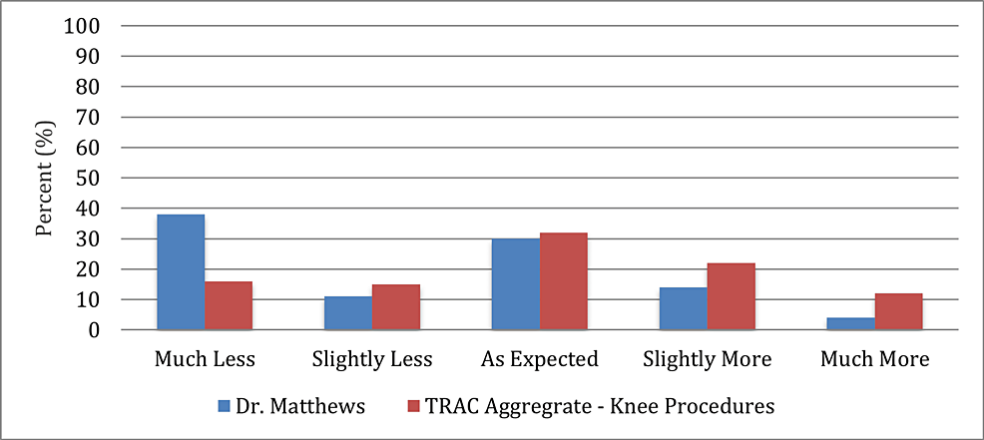
Experienced pain compared to expectations of pain.

Compared to patients that did not receive the novel-surgeon-placed block and catheter placement, these patients also had decreased pre-operative pain with 40% more patients reporting “much less pain than expected.” LOS in these patients was also reduced from two to three days to 23 hours, with one overnight stay in the hospital (Figure 5). There were no adverse effects associated with the novel surgeon-placed block or catheter placement.

## Discussion

The United States has been entrenched in an opioid overuse and abuse epidemic since before 2011. This epidemic has cost the US economy considerable human and economic capital [1,8,9,10]. It is estimated that greater than 70 million patients are prescribed opioids for postsurgical pain management annually [3]. Alarmingly, one in 15 of these patients goes on to long-term opioid use or abuse [3,4]. While there is much discussion about the primary etiology of this epidemic, it is certainly multi-factorial and beyond the scope of this paper. However, it is important to note that many new opioid users are coming from the acute care setting of which Orthopedic Surgeons are certainly playing a major role.

There has also been a significant push in the United States over the past few years to move total joint arthroplasty (TJA) into the outpatient freestanding ambulatory surgery center (ASC) arena [11-16]. This trend has only been enhanced by the recent COVID-19 pandemic. It is in this environment of the opioid epidemic and the increased interest in outpatient TJA that this investigation was initiated. Of the many challenges in establishing a successful outpatient TJA program at an ASC is post-operative pain management.

Post-operative pain management remains a major concern, as adequate pain control is one of the primary reasons for the need for inpatient care. Traditionally, pain management has relied heavily on parental and oral opioid analgesics [6]. These opioid analgesics have well-known side effects, including but not limited to nausea, emesis, dizziness, gastric distress, constipation, disorientation, respiratory depression, dependence, and addiction. Prolonged opioid use is also associated with an extended hospital LOS, and opioid-free post-operative analgesia is associated with a reduction in level of consciousness (LOC) by one to two days [16].

Significant improvements have been achieved over the past decade with regard to pain management. Most of these advancements have been related to multi-modal pain management protocols [17,18]. These protocols involve a combination of opioids and non-opioid pain medications, including NSAIDs, central, peripheral, and neuropathic pain blockers, cryotherapy, and selected regional and peripheral nerve blocks [19]. While surgeons have long performed local nerve blocks and joint capsular injection cocktails, over the past decade, regional nerve blocks have gained significant popularity [19].

Traditionally, these regional nerve blocks have been placed by anesthesia services using a nerve stimulator or, more recently, ultrasonic guidance [19,20,21-26]. One of the most popular regional blocks for TKA has been an ACB. This regional nerve block selectively blocks the saphenous nerve. The saphenous nerve is the branch from the femoral nerve that provides major sensory innervation to the knee joint. With an injection of an anesthetic agent (ropivacaine) into the adductor canal adjacent to the saphenous nerve, patients can experience excellent perioperative pain control after TKA [20,21,23]. However, anesthesia services are not always capable of or available to provide these blocks. When anesthesia is available, placement of these blocks under ultrasonic guidance requires a significant learning curve to become proficient, consistent, and efficient in providing effective anesthesia [22,24,26]. The efficacy of these blocks can vary greatly depending on the experience of the anesthesiologist and specific patient anatomy regarding body habitus and the soft tissue envelope.

While the traditional single-injection regional ACBs can provide significant relief for the first 16-24 hours, patients can experience a significant rebound of pain as the block resolves [22]. Even the newer liposomal ropivacaine agents can only provide an extended block for a maximum of 24 hours. Longer-acting (five to seven days) analgesia can be achieved though the placement of an indwelling catheter. These catheters, with a regulated controlled release of anesthetic, can provide a continuous long-acting nerve block for multiple days [22]. The placement of these indwelling catheters, however, can present challenges. Some surgeons may find that anesthesia services are either not set up to place indwelling catheters and/or not willing to provide the post-operative care or monitoring of these catheters. The placement of these catheters may also present a challenge for anesthesia services with regard to the best location for catheter placement.

These indwelling catheters are traditionally placed pre-operatively. As noted in the introduction, if placed in the mid or low (distally) adductor canal position, the catheter can invade the sterile surgical field or be in jeopardy when a tourniquet is used. In contrast, if the catheter is placed high (proximally) in the adductor canal to avoid the tourniquet and the sterile surgical field, the very real risk of a motor block on the femoral nerve is realized [22,27-30]. To address these challenges and concerns, the senior author of this study developed a novel, yet simple, safe and reproducible technique for placement of the ACB and indwelling catheter under direct visualization. Originating in the operating room (OR), this technique was further developed and studied in the cadaver lab, documenting safety and verifying efficacy. Other studies have acknowledged the possibility of saphenous nerve access through the surgical approach.

Kavolus et al. [18] published the results of their cadaveric MRI study which demonstrated a reproducible relationship between the width of the trans-epicondylar axis (TEA) and the proximal location where the saphenous nerve emerges from the adductor canal. The authors demonstrated successful dying of the saphenous nerve in eight of 11 cadaver specimens using a “blind” technique based on these measurements alone. In their paper, they concluded: “This study indicates, based on MRI measurements, cadaveric injections, and dissections, that a surgeon-performed injection of the saphenous nerve from within the knee after it exits from the adductor canal seems to be a feasible procedure. This technique may be a useful alternative to ultrasound-guided block” [18].

The senior author of this article has been successfully placing adductor canal blocks and indwelling catheters under direct visualization, using the technique described in this paper since 2016.

Limitations of this study

This study was performed retrospectively outside the setting of a clinical trial. The results of this study have not been analyzed for statistical significance, so it is unclear if the improvements in pain, opioid use, and adverse side effects are statistically significant. The findings in this article detail less than 5% of the operations that the senior author has performed with this technique. While the technique is showing promising efficacy in the pioneering surgeon's clinical practice, this article serves the purpose of exposing the community of orthopedic surgeons performing TKA to this technique while simultaneously showing promising preliminary data associated with this method. In order to fully assess the efficacy of this method in improving pain and reducing opioid usage, a proper clinical trial with appropriate analysis is warranted for further study.

## Conclusions

Using the placement technique described in this study, surgeons can reproducibly place a single injection and an indwelling catheter for a continuous nerve block in the adductor canal under direct visualization of the muscles that make up the borders of the adductor canal. This novel technique and placement have potential advantages over anesthesia placement techniques with regard to efficacy, efficiency, and safety. When using this described novel technique in combination with a comprehensive multi-modal pain management protocol, patients may experience significantly less pain, use fewer opioids, have a shorter hospital length of stay (LOS), and have less side effects without jeopardizing quality scores and patient satisfaction. There is also the potential for significant cost reduction in a bundle payment scenario with the elimination of additional anesthesia procedures.

Using the surgeon-placed adductor canal block techniques presented, in combination with a multi-modal peri-operative pain management protocol, the surgeon can address the opioid epidemic and move one step closer to establishing an outpatient total joint service. Based in part from the evidence in this study, the senior author of this paper (DEM) began a successful, same-day discharge outpatient total joint program in August 2020. At the time of submission of this manuscript, the senior author had successfully performed 403 consecutive outpatient total joint arthroplasty procedures with a mean post-operative time to discharge of 3 hours 23 minutes. To date, there have been no readmissions related to same-day discharge. At the time of submission of this manuscript, the senior author (DEM) had successfully placed indwelling catheters using this novel technique in over 1275 patients without any complications related to this placement.

## References

[1] Ben-Ari A, Chasnsky H, Rozet I (2017). Preoperative opioid use is associated with early revision after total knee arthroplasty: a study of male patients treated in the veterans affairs system. J Bone Joint Surg Am.

[2] Cancienne JM, Patel KJ, Browne JA, Werner BC (2018). Narcotic use and total knee arthroplasty. J Arthroplasty.

[3] Bendtsen TF, Moriggl B, Chan V, Børglum J (2016). The optimal analgesic block for total knee arthroplasty. Reg Anesth Pain Med.

[4] Florence C, Luo F, Rice K (2021). The economic burden of opioid use disorder and fatal opioid overdose in the United States, 2017. Drug Alcohol Depend.

[5] Almand J, Pickering T, Parsell D, Stronach B, Carlisle R, McIntyre L (2022). The successful migration of total joint arthroplasty from the hospital inpatient to outpatient ASC setting. Knee.

[6] Amara S, Adamson RT, Lew I, Slonim A (2014). Accountable care organizations: impact on pharmacy. Hosp Pharm.

[7] Alam A, Gomes T, Zheng H, Mamdani MM, Juurlink DN, Bell CM (2012). Long-term analgesic use after low-risk surgery: a retrospective cohort study. Arch Intern Med.

[8] Carroll I, Barelka P, Wang CK (2012). A pilot cohort study of the determinants of longitudinal opioid use after surgery. Anesth Analg.

[9] Cullom C, Weed JT (2017). Anesthetic and analgesic management for outpatient knee arthroplasty. Curr Pain Headache Rep.

[10] Kertkiatkachorn W, Kampitak W, Tanavalee A, Ngarmukos S (2021). Adductor canal block combined With iPACK (interspace between the popliteal artery and the capsule of the posterior knee) block vs periarticular injection for analgesia after total knee arthroplasty: a randomized noninferiority trial.. J Arthroplasty.

[11] Terkawi AS, Mavridis D, Sessler DI (2017). Pain management modalities after total knee arthroplasty: a network meta-analysis of 170 randomized controlled trials. Anesthesiology.

[12] Matthews D, Rella RT (2023). Surgeon-placed peripheral nerve block and continuous non-opioid analgesia in total knee arthroplasty is accessible intraoperatively: a cadaveric study. J ISAKOS.

[13] Florence CS, Zhou C, Luo F, Xu L (2016). The economic burden of prescription opioid overdose, abuse, and dependence in the United States, 2013. Med Care.

[14] Gardner E (1948). The innervation of the knee joint. Anat Rec.

[15] Hanson NA, Allen CJ, Hostetter LS (2014). Continuous ultrasound-guided adductor canal block for total knee arthroplasty: a randomized, double-blind trial. Anesth Analg.

[16] Lyden J, Binswanger IA (2019). The United States opioid epidemic. Semin Perinatol.

[17] Husted H, Lunn TH, Troelsen A, Gaarn-Larsen L, Kristensen BB, Kehlet H (2011). Why still in hospital after fast-track hip and knee arthroplasty?. Acta Orthop.

[18] Kavolus JJ, Sia D, Potter HG, Attarian DE, Lachiewicz PF (2018). Saphenous nerve block from within the knee Is feasible for TKA: MRI and cadaveric study. Clin Orthop Relat Res.

[19] Ilfeld BM, McCartney CJ (2017). Searching for the optimal pain management technique after knee arthroplasty analgesia is just the tip of the iceberg. Anesthesiology.

[20] Kennedy JC, Alexander IJ, Hayes KC (1982). Nerve supply of the human knee and its functional importance. Am J Sports Med.

[21] Korean Knee Society (2012). Guidelines for the management of postoperative pain after total knee arthroplasty. Knee Surg Relat Res.

[22] Lamplot JD, Wagner ER, Manning DW (2014). Multimodal pain management in total knee arthroplasty: a prospective randomized controlled trial. J Arthroplasty.

[23] Laoruengthana A, Rattanaprichavej P, Rasamimongkol S, Galassi M (2017). Anterior vs posterior periarticular multimodal drug injections: a randomized, controlled trial in simultaneous bilateral total knee arthroplasty. J Arthroplasty.

[24] Li D, Ma GG (2016). Analgesic efficacy and quadriceps strength of adductor canal block versus femoral nerve block following total knee arthroplasty. Knee Surg Sports Traumatol Arthrosc.

[25] Li D, Yang Z, Xie X, Zhao J, Kang P (2016). Adductor canal block provides better performance after total knee arthroplasty compared with femoral nerve block: a systematic review and meta-analysis. Int Orthop.

[26] Dowell D, Ragan KR, Jones CM, Baldwin GT, Chou R (2022). CDC clinical practice guideline for prescribing opioids for pain-United States, 2022. MMWR Recomm Rep.

[27] Mahoney OM, Noble PC, Davidson J, Tullos HS (1990). The effect of continuous epidural analgesia on postoperative pain, rehabilitation, and duration of hospitalization in total knee arthroplasty. Clin Orthop Relat Res.

[28] Nader A, Kendall MC, Manning DW (2016). Single-dose adductor canal block with local infiltrative analgesia compared with local infiltrate analgesia after total knee arthroplasty: a randomized, double-blind, placebo-controlled trial. Reg Anesth Pain Med.

[29] Paul JE, Arya A, Hurlburt L, Cheng J, Thabane L, Tidy A, Murthy Y (2010). Femoral nerve block improves analgesia outcomes after total knee arthroplasty: a meta-analysis of randomized controlled trials. Anesthesiology.

[30] Pham Dang C, Gautheron E, Guilley J (2005). The value of adding sciatic block to continuous femoral block for analgesia after total knee replacement. Reg Anesth Pain Med.

